# Determining Candidate Hypobaric Hypoxia Profiles for Humane Killing of Laboratory Mice

**DOI:** 10.3389/fvets.2022.834478

**Published:** 2022-03-23

**Authors:** Jasmine M. Clarkson, Dorothy E. F. McKeegan, Julian Sparrey, Francesco Marchesi, Matthew C. Leach, Jessica E. Martin

**Affiliations:** ^1^Institute of Biodiversity, Animal Health and Comparative Medicine, College of Medical Veterinary and Life Sciences, University of Glasgow, Glasgow, United Kingdom; ^2^Livetec Systems Ltd., Bedford, United Kingdom; ^3^School of Veterinary Medicine, College of Medical Veterinary and Life Sciences, University of Glasgow, Glasgow, United Kingdom; ^4^School for Natural and Environmental Sciences, Newcastle University, Newcastle upon Tyne, United Kingdom; ^5^The Royal (Dick) School of Veterinary Studies, The Roslin Institute, The University of Edinburgh, Edinburgh, United Kingdom

**Keywords:** laboratory rodent, low atmospheric pressure, hypobaric hypoxia, animal welfare, euthanasia

## Abstract

Millions of mice are used annually in scientific research and must be humanely killed. Despite significant welfare concerns, carbon dioxide exposure remains the most common killing method, primarily because there is no practical and humane alternative. We explored whether hypobaric hypoxia *via* gradual decompression could induce a non-recovery state in anesthetized male C57BL/6 and Balb/c laboratory mice. We aimed to determine if this was possible in a feasible timescale with minimal pathological consequences, as a proof-of-principle step. Systematic evaluation of two decompression rates (75, 150 ms^−1^) and three profile shapes (accelerated, linear, gradual) in a factorial design revealed that hypobaric hypoxia effectively induced a non-recovery state in anesthetized laboratory mice, irrespective of decompression rate and shape. Mice took longer to reach a non-recovery state with the 75 ms^−1^ decompression rate (75 ms^−1^: 257 ± 8.96 vs. 150 ms^−1^: 214 ± 7.26 s), with longer latencies in gradual and linear shaped profiles. Accelerated shaped profiles were least susceptible to meaningful refinement *via* rate. The only pathological changes of concern were moderate middle ear congestion and hemorrhage. These findings suggest that hypobaric hypoxia has potential, and subsequent work will evaluate the welfare consequences of gradual decompression in conscious mice, to identify decompression profiles that minimize welfare harms associated with ear barotrauma.

## Introduction

The ethical justification for the use of animals in research relies on the prevention of unnecessary harms, minimization of necessary harms and the optimization of net benefits. An important pre-requisite of ethical approval is for animals to be humanely killed upon completion of their scientific use or breeding program, which in the UK, is regulated under Schedule 1 of the Animals Scientific Procedures Act 1986 (1). Schedule 1 methods are expected to be the most humane and as such are used as endpoints to prevent unnecessary suffering; indeed, these methods are often referred to as “euthanasia” rather than killing.

Laboratory mice remain the most widely used model organism for biomedical research (2). In the UK alone, ~2.5 million mice are used for scientific purposes each year, including direct use in experimental procedures and indirect use for the creation of transgenic mice (2). Of the commonly used and generally permitted killing methods for laboratory rodents, exposure to a rising concentration of carbon dioxide (CO_2_) gas remains the most common choice given its practical advantages, compatibility with high throughput and non-contact nature (3, 4). However, there are significant welfare concerns surrounding its use. Over recent years, considerable evidence of aversion to CO_2_ has been reported, including the induction of anxiety, dyspnoea, and pain at high concentrations (4–8). During CO_2_ exposure, rodents show behaviors indicative of fear and anxiety at concentrations as low as ~10% (9, 10), which is significantly lower than the concentrations required for loss of consciousness (around 30%) (11, 12). While there is some controversy surrounding the severity of the affective state consequences of exposure to CO_2_ (3), there is general agreement that CO_2_ inhalation is aversive and there is a requirement for the development of high-throughput alternatives (3, 4, 13, 14). As a result, its inclusion (and indirect endorsement) in existing legislation and guidance is of significant concern, not only from the perspective of animal welfare, but also risks damaging public acceptance of animal research. Therefore, there is an urgent unmet requirement to find a practical alternative to CO_2_ with better welfare outcomes.

Gradual decompression describes a progressive reduction in air pressure, simulating a process equivalent to ascending to a high altitude. High altitudes are associated with low atmospheric partial pressures of atmospheric gases, including oxygen (O_2_), resulting in hypobaric hypoxia which ultimately leads to unconsciousness and death. There has been growing interest in recent years in the potential of hypobaric hypoxia *via* gradual decompression to provide a humane killing method for non-human animals in both agricultural and laboratory settings, especially since the onset of symptoms are insidious in humans presenting with no obvious signs of pain and/or discomfort (15–17). A comprehensive welfare assessment previously showed that gradual decompression, commercially referred to as Low Atmospheric Pressure stunning (LAPS), is humane for use in chickens, and as such it was added to the EU slaughter regulations in 2018 (18).

It remains unknown whether gradual decompression could offer an effective and reliable method for killing laboratory mice. Many rodent species, including mice, appear to have not only evolved a sensitivity to CO_2_ (19) but also to reduced O_2_ concentrations in inspired air, likely due to their natural requirement to burrow and the associated risk of hypoxia (20). Evidence from approach avoidance paradigms has demonstrated that rats left a chamber at a mean O_2_ concentration of 6.8% (21) and mice at 8.6% (22). There are few studies focused on hypoxic killing of rodents and they are all based on the exposure to inert gases, such as argon and nitrogen (normobaric hypoxia). However, these have not been fruitful in offering a humane alternative to exposure to CO_2_ (4, 23–25). Although of relevance, these studies have been based on the displacement of O_2_ with high concentrations of inert gases under normobaric conditions. This is unlike gradual decompression which leads to hypoxia under hypobaric conditions, whereby the percentage of O_2_ within the air remains constant, but instead decreases in atmospheric pressure lead to reductions in the partial pressure of O_2_ (26)_._ In humans, a number of physiological responses (e.g., ventilation) are different between equivalent hypobaric and normobaric hypoxia, and proposed mechanisms for this include increased alveolar dead space, and changes in chemosensitivity (27). Importantly, unlike the displacement of air with inert gases, decompression is more easily controllable and can be reliably achieved gradually over chosen time periods and with variable rates within a single cycle, allowing for a wide range of decompression profiles. As such, the finding that a single exposure to 98% argon causes a conditioned place preference in mice (4), is of limited relevance when predicting responses to gradually induced hypobaric hypoxia. In humans, it is well known that exposure to high altitudes can lead to progressive loss of cognitive and psychomotor skills before loss of consciousness and death (15–17, 28, 29). Furthermore, it is considered especially dangerous for air crew during loss of cabin pressure because acute progressive hypoxia is often insidious, presenting with no obvious signs of pain or discomfort before loss of consciousness (16, 17, 29). However, the rate of decompression is likely to be directly related to its welfare impact during the conscious phase of killing. If the rate of decompression is explosive (<1 s to near vacuum) or rapid (several seconds to very low vacuum) severe organ damage and hemorrhage may occur (30), which are clearly unacceptable from both a welfare and scientific perspective. If the final pressure is too low (above Armstrong's line; 19,202 m), and is achieved rapidly, the production of bubbles of water vapor in the blood and joint fluids may cause mechanical damage of organs and tissues and have associated negative welfare outcomes (31). However, this can be avoided given that the equivalent maximum altitude needed to achieve death is significantly below Armstrong's line (32). Nonetheless, given that “gradual” decompression encompasses a wide range of possible rates (i.e., anything slower than several seconds), potentially painful sensations due to expansion of trapped gases in the respiratory system, alimentary tract, sinuses of the skull and middle ear are still possible and must be fully explored (33).

The decompression rates established for use in poultry are similar to those observed in humans during flight at high altitudes in unpressurised cabins (34). When describing decompression, equivalent altitude is often used, for example ambient barometric pressure at sea level (0 m) is 101.3 kPa (1,013 mBar). In commercial LAPS, an equivalent altitude of 8,459 m (33.3 kPa; 333 mBar) is reached at an average rate of 127 ms^−1^ during the first ascent phase (35). However, there are notable differences between mammalian and avian anatomy and physiology, which could affect responses to hypobaric hypoxia. Given their ability for flight at high altitudes, birds have numerous adaptations for enhanced oxygen delivery such as; high hemoglobin oxygen affinity, large lung to blood volume ratios and tubular arrangement of gas exchange systems unlike the spherical alveoli of mammals (36). Rodents are particularly resistant to death *via* both normobaric and hypobaric hypoxia (37–40) therefore systematic evaluation of rates capable of death with minimal pathological consequences is necessary as an initial proof of principle step. We systematically explored two rates (75 and 150 ms^−1^) of decompression, to reach an equivalent altitude of approximately 11,800 m (20 kPa; 200 mBar), with decompression profile selection based on previous non-harmful rates observed in humans and non-human animals (33, 34, 41). We characterized their effects on behavior and pathological consequences in laboratory mice, whilst protecting welfare using terminal general anesthesia. Unlike LAPS in a commercial slaughter setting, which serves as a stunning method before exsanguination, our cycle times were not constrained by food chain considerations (33, 34). This provided an opportunity to manipulate not only overall decompression rate, but also the degree of pressure changes within the cycle using multiple stepwise phases to manipulate the decompression profile shape. Using a factorial design (two rates and three shapes), we systematically evaluate behavioral and pathological responses to decompression profiles and concluded whether they were candidates for humanely inducing a non-recovery state in two laboratory mouse strains (C57BL/6 and Balb/c).

## Methods and Materials

### Ethical Statement

All experiments were conducted at the University of Glasgow following ethical approval from the Animal Welfare and Ethical Review Body (AWERB) and project license approval from the Home Office (PPL:TP900S002; Protocol 1). Experiments were fully compliant with the EU Directive (2010/63/EU) and Animals Scientific Procedures Act (1986). All methods are reported in accordance with ARRIVE guidelines for the reporting of animal experiments (42).

### Animals, Housing, and Husbandry

Seventy-two male mice from two laboratory strains (36 C57BL/6 and 36 Balb/c) were obtained from Charles River UK and were delivered at ~8 weeks of age across two batches (Arrival dates; batch 1: 22/09/20, batch 2: 03/11/20). Mouse strain selection was to represent two of the most widely used laboratory mouse strains in biomedical research, as well as focusing on male mice initially, given their widespread use across research models. Mice were housed in same strain triplets in filter top caging (1,284 L, Techniplast, London, UK) with eco-pure aspen sawdust bedding (Datesand Ltd. Manchester, UK), nesting substrate (sizzlenest, Datesand Ltd.), a clear Perspex tunnel (Datesand Ltd.) with food (BK001, Special Diet Services, Essex, UK) and water available *ad libitum*. Mice were obtained from Charles River UK with Specific Pathogen Free (SPF) health status in accordance with FELASA health monitoring recommendations (43). All animals acclimatized for 1 week prior to commencement of experimental work and weighed 25.1 ± 0.2 g (Min: 21.4 g; Max: 29.5 g) at the time of experimental work, with no difference in weight between the two strains [C57BL/6: Mean 24.9 ± 0.2 g (Min: 22 g; Max; 27.3 g); Balb/c: Mean 25.3 ± 0.3 g (Min: 21.4 g; Max: 29.5 g)]. All mice were handled using refined tunnel handling techniques to mitigate against background stress and anxiety (44, 45). All animals were checked daily, and no adverse effects were reported.

### Anesthesia

Prior to decompression exposure, mice were anesthetized with subcutaneous administration of ketamine/xylazine hydrochloride [C57BL/6 dosage: 100 mg/kg ketamine and 10 mg/kg xylazine; Balb/c dosage: 125 mg/kg ketamine and 12.5 mg/kg xylazine (46)]. The animal was continuously monitored, and anesthetic depth was assessed at 5 min intervals following loss of the righting reflex until a surgical plane of anesthesia was reached [defined as loss of several reflexes including the pedal withdrawal reflex (toe pinch), corneal reflex and whisker twitch in addition to a general loss of muscle tone (46)]. Induction to surgical plane had a mean (±SE) duration of 17 min 26 ± 36 s (Min: 9 min 51 s, Max: 48 min 54 s). Mice only underwent gradual decompression once a surgical plane of anesthesia was confirmed, to ensure depth and duration of anesthesia were maintained throughout. All mice were continuously behaviorally and physiologically monitored during decompression. As expected, no behavioral or physiological indicators of consciousness were observed.

### Exposure to Gradual Decompression

Mice were randomly assigned *via* random number generator (random.org) to one of six decompression profiles according to a randomized-block factorial design using a Latin square and were exposed to gradual decompression individually. Blocking factors included cage [to ensure all three animals were killed on the same day to reduce and mitigate against social isolation and stress from remaining in the home cage overnight (47, 48)] and batch. In batch 1, six mice were killed per day across six consecutive days, whereas in batch 2, nine mice were killed per day across four consecutive days. This design was fully balanced with respect to strain and decompression profile. In error, decompression profile linear 150 ms^−1^ was applied to one Balb/c mouse instead of a linear 75 ms^−1^ profile.

Once a surgical plane of anesthesia was confirmed, each mouse was transferred into the decompression chamber and positioned in the supine position on the Vetbed^®^ covered floor of the box. ECG electrodes were attached (vet-tech, Congleton, UK, ERM8010) for real-time monitoring of cardiac activity. The decompression chamber was custom designed (Livetec Systems Ltd., Bedford, UK) and made from transparent acrylic (30 mm thick) with external dimensions including attachments of 610 mm (W) × 610 mm (D) × 470 mm (H) and internal dimensions of 400 mm (W) × 400 mm (D) × 400 mm (H) (Supplementary Figure S1). The chamber connects to an automated programmable logic controller (PLC) system from which fully flexible programming of parameters are controlled to achieve candidate decompression profiles using a touch screen user interface. The chamber is connected to a vacuum pump (DVP lubricated rotary vane vacuum pump, LC25 2018) by hose and proportional control valve to enable fixed decompression rates, adjustable according to cycle time or pressure thresholds (outlined fully in Table 2). In line with previous work (34, 35), decompression profiles consisted of two distinct phases. Phase 1 involved decompression to a target pressure of 200 mBar (~11,800 m equivalent altitude) followed by a subsequent minimum hold phase (phase 2) for 5 min, which was extended in additional 1-min increments until confirmation of death (i.e., >30 s of motionless) as necessary. Following each cycle, recompression was facilitated manually over a period of at least 3 min (222 ± 2.26 s) by an air admittance valve, monitored with a vacuum gauge. Pressure change was controlled manually by placement of a marker on the air admittance valve to maintain consistent recompression between individuals and was timed using a stopwatch. This ensured consistent, slow recompression at an overall average rate that was no faster than the most rapid decompression profile. Chamber pressure, temperature and humidity were recorded every second using precision probes during baseline, decompression and recompression phases of the cycles.

### Behavioral Observations

The behavior of each mouse was video recorded using a GeoVision surveillance system (GV800B) connected to four wired cameras (Bird box camera 1,080 p with IR Night vision) sitting at different positions outside of the decompression chamber, providing a full bilateral cranial-caudal view. The system allowed direct live monitoring of the mouse on an external computer screen and captured footage for analysis. Frequency of urination was noted live, at the time of killing and confirmed using recorded behavioral footage. Behavioral footage for each mouse was observed using Noldus Observer XT (version 12) by a single observer who was blinded to decompression profile but not to mouse strain (impossible given their different coat colors). An ethogram (Table 1) focused on measures indicative of hypoxia and death was developed in line with previous work on gaseous euthanasia methods in laboratory mice (23, 49–51). Behavioral variables measured included latencies, counts, rates and total durations. Data was exported from Observer to Microsoft Excel.

**Table 1:** Ethogram showing behavioral latencies, counts, durations and rates recorded.

**Behavior**	**Description**	**Measures**
Gasp	Rapid deep inspiration of breath that is not agonal or part of normal rhythmic breathing. This may be associated with opening of the mouth.	Latency Counts Rate
Agonal gasp	Very deep inspiration often with opening of the mouth that is accompanied by diaphragm movement and lifting of the head.	Latency Counts
Cessation of rhythmic breathing	The point at which breathing at regular rate ceases. Indicated by lack of body wall movement and a lack of movement of the diaphragm.	Latency
Fore limb movement	Any movement including twitching of the right or left forelimbs.	Latency Duration
Hind limb movement	Any movement including twitching of the right or left hind limbs.	Latency Duration
Head movement	Any movement including twitching of the head or neck that is not a consequence of agonal gasps.	Latency Duration
Tail movement	Any movement including twitching of the tail.	Latency Duration
Urination	The release of urine from the body, either under passive or active control.	Latency
Motionless	No discernible body or breathing movements including the absence of heart flutter. Conservative marker of death.	Latency

*Note that since the mice were terminally anesthetized, behaviors are limited to reflexive and postural and do not infer the welfare status of the animals*.

### Post-mortem and Histological Assessment

Following recompression all mice were dead (100% kill success) and upon removal from the chamber, death was immediately confirmed by absence of reflexes, respiration and cardiac activity and formally confirmed by severing the femoral artery (permanent cessation of the circulatory system) in accordance with Schedule 1 of A(SP)A (UK). A subset of mice (*n* = 4 per strain and decompression profile, 48 in total) were transferred to a separate room for full post-mortem examination. Macroscopic examinations were conducted by a single veterinary pathologist (FM) who was blinded to decompression profile.

### Semi-quantitative Scoring of Macroscopic Changes

Prior to post-mortem dissection and following removal of the ear pinna, the tympanic membranes were assessed by inspection of the external ear canals with a stereomicroscope and the tympanic membranes were noted as intact or ruptured.

During post-mortem dissection the following changes were assessed: presence of blood (hemorrhage) from the oral cavity, external ears and nostrils; presence of red discolouration suggestive of congestion/hemorrhage in the subcutis and superficial fascia of the cervical/submandibular region and on the surface of the skull in the occipital region and at the level of the biceps femoris muscles; presence of red fluid at the level of the stifle joints; red discolouration suggestive of congestion/hemorrhage visible from external inspection of the stomach, small and large intestine, pancreas, thymus, heart, liver and kidneys, areas of purple/dark red discolouration (suggestive of congestion/hemorrhage and/or atelectasis) in the right and left pulmonary lobes. Additional assessments included dilation of the stomach, intestine and enlargement of the spleen. The findings listed above were scored with the following semi-quantitative grading scale: 0 = absent; 1 = minimal; 2 = mild; 3 = moderate; 4 = marked; 5 = severe. The presence of alimentary content in the lumen of the stomach was recorded with the following semi-quantitative grading scale: 0 = stomach empty; 1 = small amount; 2 = moderate amount; 3 = large amount (see Supplementary Table S1).

### Histological Assessment

Following 48–72 h of fixation in 10% neutral buffered formalin, the lungs and heads were processed for histological assessment. Following immersion for 48 h in a decalcifying solution (65% sodium citrate 20 and 35% formic acid) the head specimens were trimmed transversally at multiple levels to expose the middle and inner ear structures, the retrobulbar space and tissues, and the maxillary sinus. Lungs and head samples were routinely processed to paraffin blocks in an Excelsior AS processor (Thermo Scientific). From the paraffin embedded tissues 3 μm thick sections were mounted on charged glass slides. Sections were dewaxed in Histoclear (D-limonene), rehydrated in descending alcohols, rinsed in distilled water, and stained with Haematoxylin and Eosin (H&E) according to standard protocols (52). Sections were finally dehydrated in ascending alcohols and Histoclear and cover-slipped.

Tissue sections were examined by a veterinary pathologist (FM) under a BX53 Olympus microscope. The following microscopic findings were assessed in the lungs (right lobes and left lobe examined separately): congestion of vessels and septal capillaries, hemorrhage (defined as accumulation of extravasated red blood cells in alveolar spaces and/or perivascular spaces) and atelectasis (refer to Supplementary Material for representative images). The following microscopic findings were assessed for congestion/hemorrhage in the sections of the head: the mucosa of the maxillary sinus, the orbital retrobulbar soft tissues, the middle ear (defined as ectactic vessels engorged with blood and/or presence of extravasated red blood cells in the mucoperiosteum of the tympanic bulla, with presence of extravasated red blood cells in the lumen of the tympanic bulla), and inflammation of the middle ear (defined as presence of inflammatory cells in the mucoperiosteum and/or in the lumen of the tympanic bulla). Microscopic changes were scored with the following semi-quantitative grading scale: 0 = absent; 1 = minimal; 2 = mild; 3 = moderate; 4 = marked; 5 = severe (refer to Figure 5 for representative images). Histological evidence of discontinuity of the tympanic membrane, suggesting rupture, either unilateral or bilateral, was assessed as 0 = absent or 1 = present. Representative microphotographs were taken with a SC100 digital camera (Olympus) connected with the microscope and interfaced with a cellSens image capture software (Olympus).

### Statistical Analyses

All statistical analyses were conducted in R (v 4.0.3, R Core Team) (53) *via* the R Studio platform (Version 1.3.1093, RStudio, Boston, USA, PBC, 2009-2020). All data was collated and processed within R using the tidyverse package (54). All graphical summaries were created using the ggplot2 package (55). Behavioral and physiological data were analyzed with generalized linear mixed models (GLMMs) *via* the glmmTMB package (56) and general linear mixed models (GLMs) *via* the lme4 package (57) were used to identify factors which may affect each behavioral and pathological measure. Model fit was determined by examination of residuals *via* the DHARMa package (58) and appropriate error distributions set for GLMMs. Ordinal pathological data were analyzed using Cumulative Link Mixed Models (CLMMs) [packages: ordinal (59) and RVAideMemoire (60)] to compare congestion and hemorrhage scores (0–5) in organs and tissues with the threshold set to equidistant. CLMMs were assessed *via* nominal and scale test functions in the ordinal package (61). All models included fixed factors of decompression rate (2 levels: 75 ms^−1^ or 150 ms^−1^), profile shape (3 levels: accelerated, gradual or linear) and strain (2 levels: C57BL/6 or Balb/c). Interactions between fixed factors were included in the model. Relative humidity and temperature were included in the model as covariates to ensure they did not have a significant effect upon outcome variables and removed if non-significant to improve model fit (62). All behavioral models included mouse weight and the unique cage number nested within batch as random effects to account for weight differences and non-independence of mice from the same cage. Statistical significance based on *p* < 0.05 threshold were calculated using the ANOVA function [car package (63)] to ascertain differences derived from fixed effects and interactions, and only statistically significant results are reported. Pairwise comparisons were reported using estimated marginal means *via* the emmeans package, with *P*-values adjusted for multiple comparisons using the Tukey method (64).

## Results

**Figure 1:**
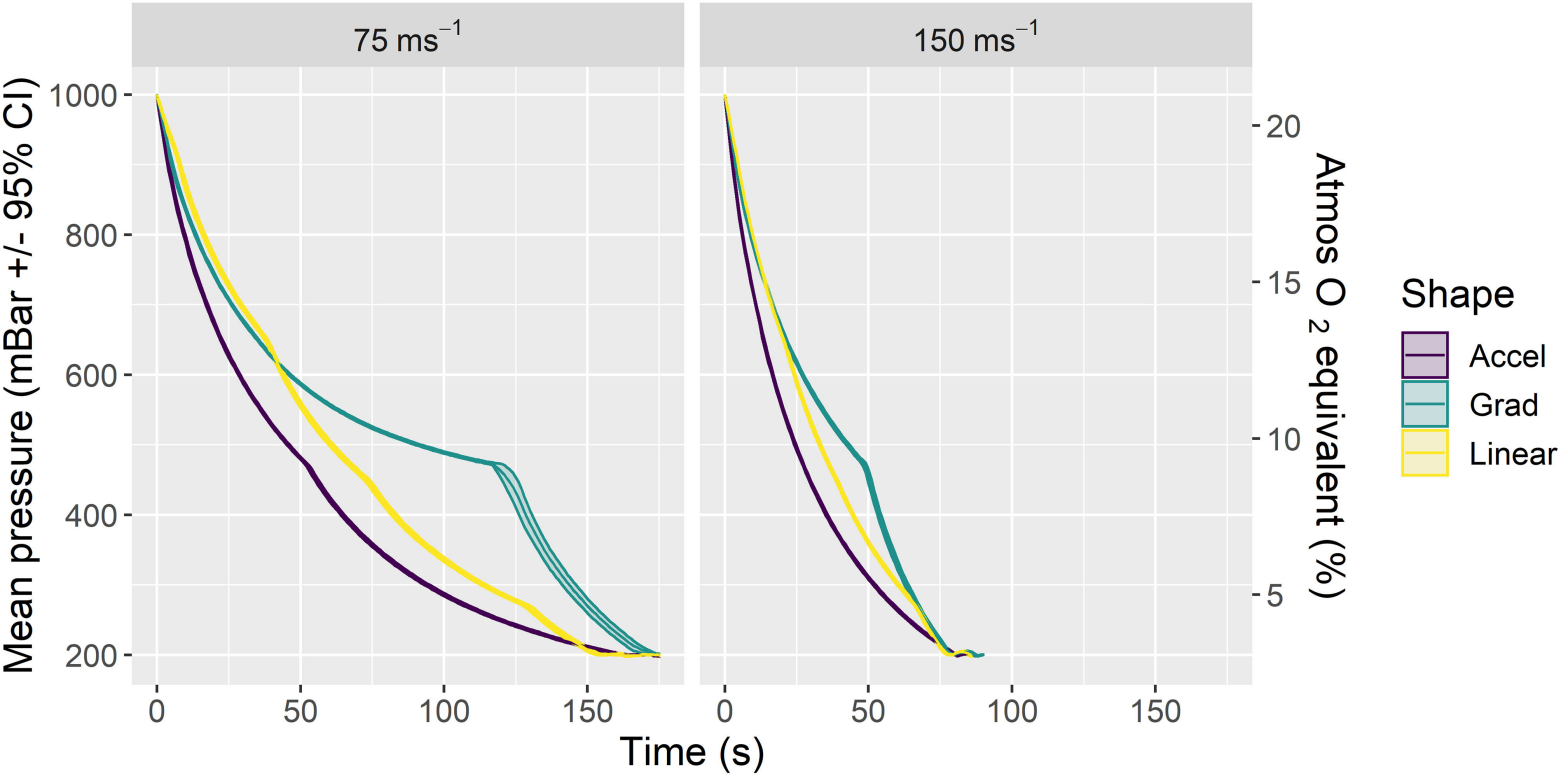
Mean ± 95% CIs pressure (mBar) for phase 1 of the six candidate decompression profiles assessed according to rate (left panel: 75 ms^−1^, right panel: 150 ms^−1^) and shape (Accelerated (Accel), Gradual (Grad) and Linear). Each profile consisted of gradual decompression to the target pressure of 200 mBar (~11,800 m equivalent altitude) in ~157 s (75 ms^−1^ rate) or 79 s (150 ms^−1^ rate) for all shapes. Once target pressure was reached, a subsequent minimum hold phase for 5 min (phase 2) was initiated and extended in additional 1-min increments until death confirmation (i.e., >30 s of motionless) as necessary. The chamber was then recompressed (phase 2 and recompression not displayed in figure, details in Table 2). The secondary y axis (right) is a scaled indication of comparable atmospheric O_2_ equivalent as a percentage of inspired air at sea level, using a saturated water vapor value of 6.3 kPa.

We examined two average decompression rates (75 and 150 ms^−1^) expressed through three profile shapes within each rate (accelerated, linear and gradual) resulting in six candidate decompression profiles (Figure 1; Table 2) in two strains (C57BL/6 and Balb/c) of terminally anesthetized mice. Figure 1 shows phase 1 of decompression profiles only, illustrating the consistency of gradual decompression. Gradual decompression was effective in evoking a non-recovery state in all mice, demonstrating 100% kill success irrespective of decompression rate and shape. Unless stated, we found no significant effects of mouse strain.

**Table 2:** Outline of the six decompression profiles according to rate and shape.

**Decompression profile [shape and target rate (ms^−1^)]**	***n***	**Average decompression time (±SE) (min–max) to 200 mBar** **(Phase 1)**	**Achieved rate range (~ms^−1s^)**	**Average cycle length** **(±SE) (min–max)** **(Phase 1 and 2)**	**Average temperature change (°C) (±SE) (Phase 1)**	**Average relative humidity change (%) (±SE)** **(Phase 1)**
Accelerated 75	12	164.50 ± 0.65s (161–169 s)	69.8–73.3	479.5 ± 15.1s (461–645 s)	−3.40 ± 0.07	−30.1 ± 1.0
Linear 75	11	155.18 s ± 0.62s (152–159 s)	74.2–77.6	482.0 ± 26.8s (452–750 s)	−3.58 ± 0.19	−29.7 ± 1.3
Gradual 75	12	171.83 ± 1.14s (166–178 s)	66.3–71.1	607.4 ± 135.7s (467–2,100s^*^)	−3.63 ± 0.13	−28.9 ± 0.9
Accelerated 150	12	81.67 ± 0.26s (81–84s_	140.5–145.7	396.5 ± 14.8s (381–559s)	−4.25 ± 0.11	−30.1 ± 1.1
Linear 150	13	78.69 ± 0.43s (76–81 s)	145.7–155.2	387.9 ± 9.0s (377–496 s)	−4.14 ± 0.13	−29.9 ± 1.0
Gradual 150	12	79.58 ± 0.43s (77–82 s)	143.9–153.2	379.7 ± 0.4s (378–382 s)	−4.41 ± 0.07	−29.5 ± 1.4

*This includes decompression time, which includes the time taken to reach the target pressure of 200 mBar (Phase 1) which was followed by a subsequent hold period (Phase 2). Rates reported here represent the overall average decompression rate calculated as an equivalent ascension rate (ms^−1^). Average changes in temperature (°C) and relative humidity (%) during phase 1 of decompression (from cycle start to 200 mBar).*

* *Maxima reflects outlier for one mouse undergoing the gradual 75 profile whereby cycle length was extended due to ongoing abdominal twitching*.

### Behavioral Findings

**Figure 2:**
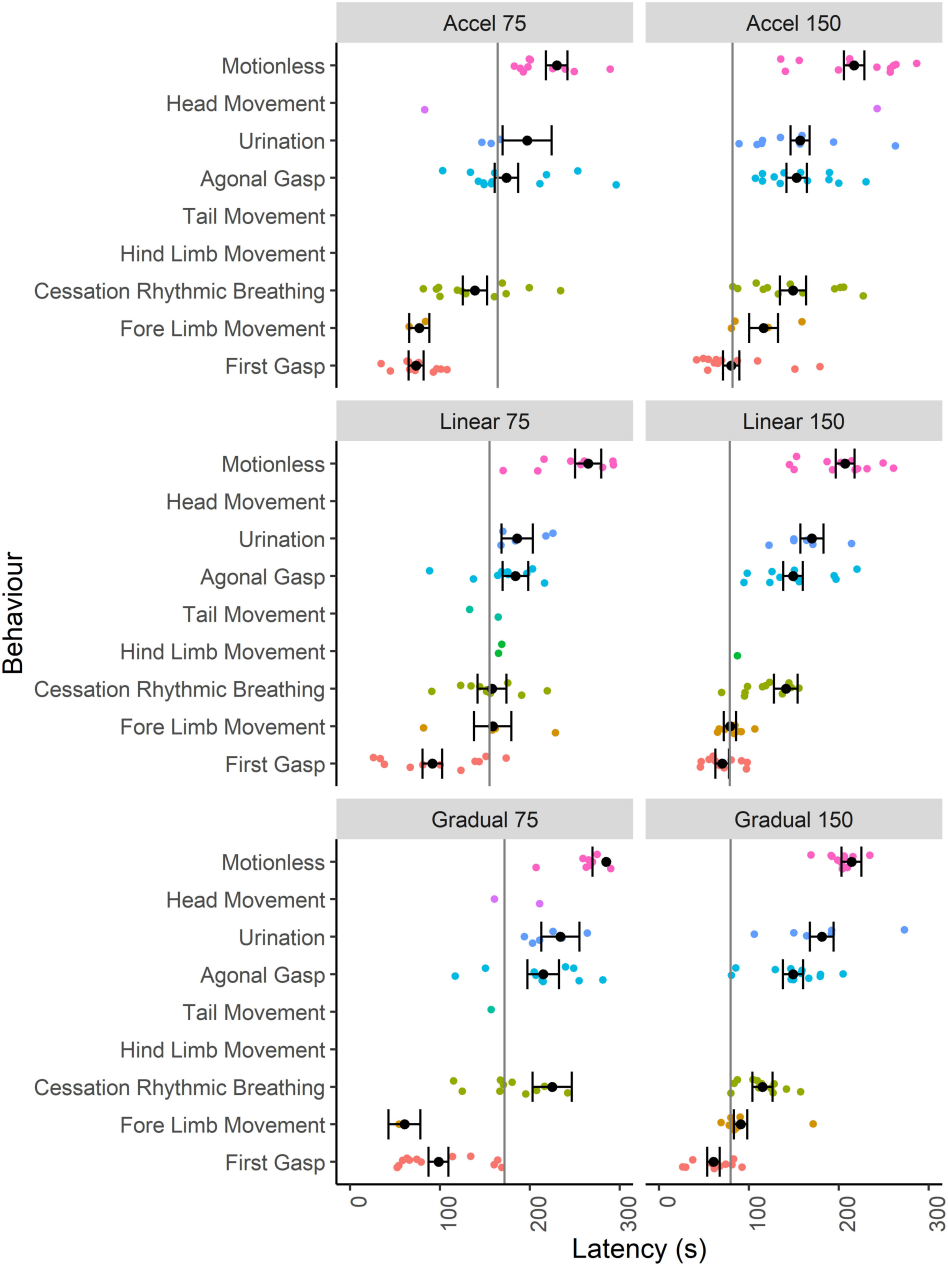
Individual data points and mean (±SE) latencies (s) of key reflexive behaviors relating to respiratory responses (e.g., gasp) and non-recovery indicators (e.g., motionless) observed across the six candidate decompression profiles according to both rate (75 and 150 ms^−1^) and shape [Accelerated (Accel), Linear and Gradual, *n* = 12 per profile, *n* = 13 linear 150 ms^−1^, *n* = 11 linear 75 ms^−1^]. The vertical gray lines represent mean latencies to reach 200 mBar and the start of the Phase 2 hold period for each decompression profile.

**Figure 3:**
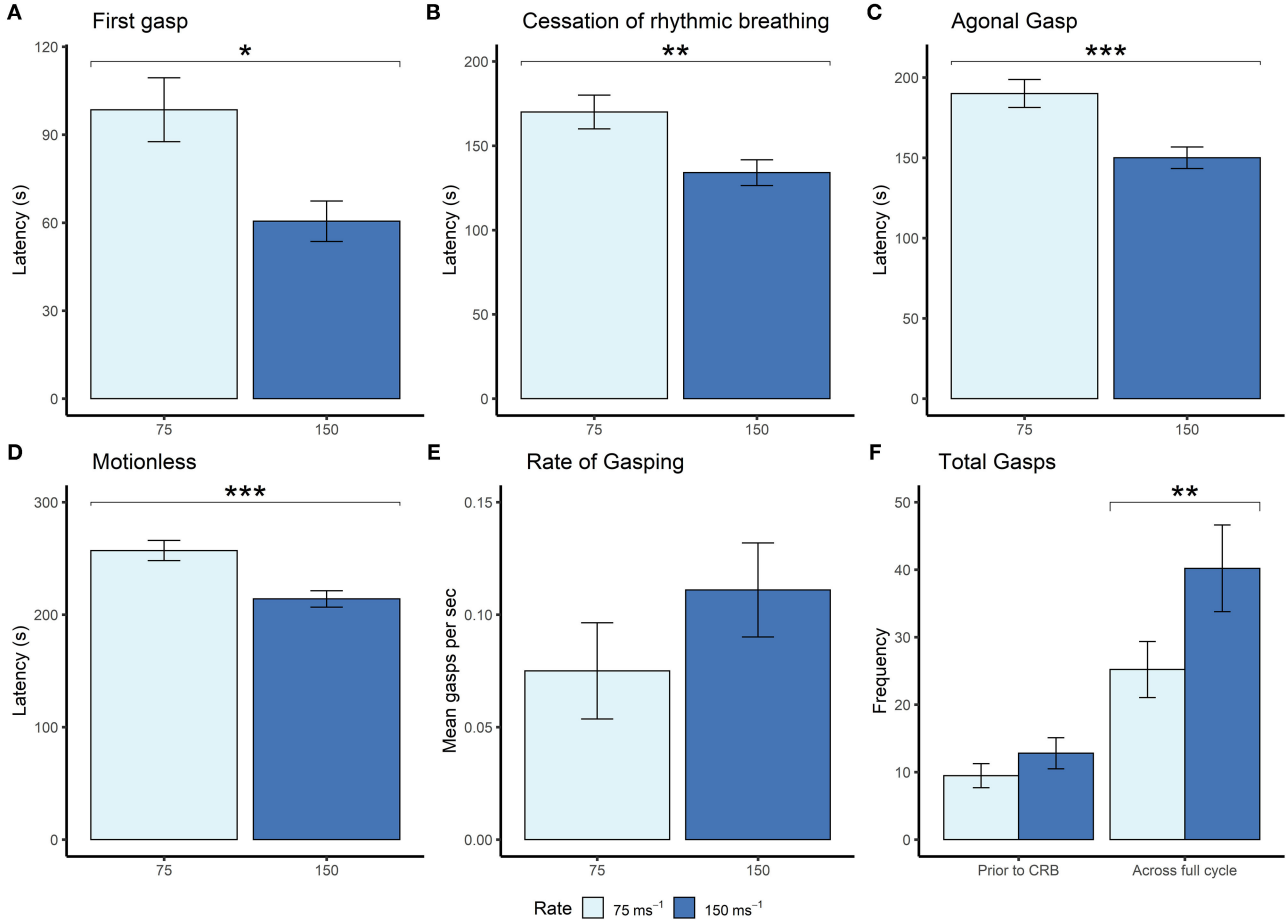
Mean (±SE) **(A)** latencies to first gasp (75 ms^−1^ *n* = 35; 150 ms^−1^ *n* = 37), **(B)** latencies to cessation of rhythmic breathing (75 ms^−1^ *n* = 35; 150 ms^−1^ *n* = 37), **(C)** latencies to agonal gasping (75 ms^−1^ *n* = 33; 150 ms^−1^ *n* = 37), **(D)** latencies to motionless (75 ms^−1^ *n* = 34; 150 ms^−1^ *n* = 37), and **(E)** rate of gasping (frequency per second) prior to cessation of rhythmic breathing, and **(F)** frequencies of gasps occurring prior to cessation of rhythmic breathing (prior to CRB) and the whole decompression cycle length (across full cycle; 75 ms^−1^ *n* = 35; 150 ms^−1^ *n* = 37) according to overall decompression rate [75 ms^−1^ (light blue) and 150 ms^−1^ (dark blue)]. *p < 0.05, **p < 0.01, ***p < 0.001.

Given that the mice were terminally anesthetized, behavioral output was limited and consisted of unconscious and reflexive movements. Observed behavioral responses followed a generally consistent sequence of events (first gasp, fore limb movement, cessation of rhythmic breathing, agonal gasping, urination and motionless) irrespective of decompression profile (Figure 2). We found significant effects of decompression rate on several behavioral measures, with the slower rate resulting in elongated latencies and cessation of behaviors, as expected (Figures 3A–D). These included mean (±SE) latencies to first gasp [*t*_(58)_ = 2.533, *p* = 0.014], cessation of rhythmic breathing [*t*_(59)_ = 3.098, *p* = 0.003], agonal gasping [*t*_(56)_ = 3.874, *p* < 0.001], and time to motionless/death [*t*_(55)_ = 4.964, *p* < 0.001]. Furthermore, we found significant effects of decompression rate when analyzing the total number of gasps occurring across the whole cycle length [*t*_(58)_ = −2.902 *p* = 0.005; Figure 3F], with the 75 ms^−1^ decompression rate resulting in fewer gasps. However, there was no difference in those occurring prior to the cessation of rhythmic breathing [*t*_(59)_ = −1.531, *p* = 0.131; Figure 3F]. There was no effect of decompression rate on the rate of gasping prior to the cessation of rhythmic breathing [*t*_(57.5)_ = −1.650, *p* = 0.104; Figure 3E].

**Figure 4:**
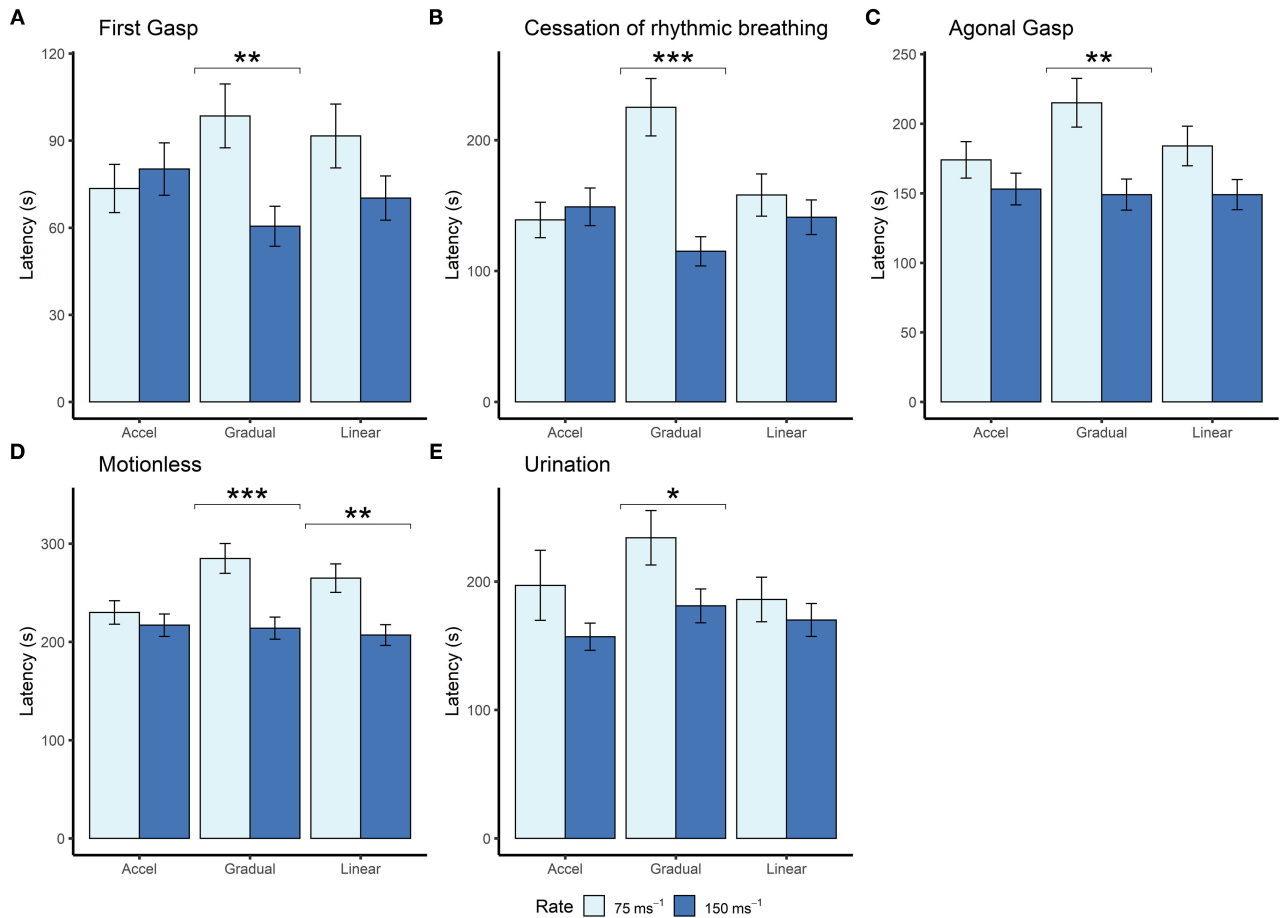
Mean (±SE) latencies to **(A)** first gasp (*n* = 12 per profile, *n* = 13 linear 150ms^−1^, *n* = 11 linear 75 ms^−1^), **(B)** cessation of rhythmic breathing (*n* = 12 per profile, *n* = 13 linear 150 ms^−1^, *n* = 11 linear 75 ms^−1)^, **(C)** agonal gasping (*n* = 12 per profile, *n* = 13 linear 150 ms^−1^, *n* = 11 linear 75 ms^−1^, *n* = 10 gradual 75 ms^−1^), **(D)** motionless (*n* = 12 per profile, *n* = 13 linear 150 ms^−1^, *n* = 11 linear 75 ms^−1^, *n* = 11 gradual 75 ms^−1^), and **(E)** urination (linear 75 ms^−1^ *n* = 5; linear 150 ms^−1^ *n* = 6; accelerated 75 ms^−1^ *n* = 3; accelerated 150 ms^−1^ *n* = 9; gradual 75 ms^−1^ *n* = 6; gradual 150 ms^−1^ *n* = 6) according to decompression rate [75 ms^−1^ (light blue) and 150 ms^−1^ (dark blue)]. *p < 0.05, **p < 0.01, ***p < 0.001.

Although we found no effects of profile shape independent of rate, within both the gradual and linear shape profiles the rate of decompression impacted several behavioral measures (longer latencies observed for the 75 ms^−1^ rate compared to the 150 ms^−1^ rate), which was never the case for the accelerated shape. This interaction was apparent when examining latencies for first gasp, agonal gasping, motionless, cessation of rhythmic breathing and urination (Figure 4). Taken together, these data suggest that for the accelerated shape, the overall mean rate of decompression had no effect on key behavioral outcomes at the rates tested.

Examining the effect of shape within each rate separately revealed that shape had an independent effect within the 75 ms^−1^ but not in the 150 ms^−1^ rate for some behavioral measures. Mice took longer to cease rhythmic breathing in the gradual 75 ms^−1^ (225 s ± 21.9) profile compared to both accelerated 75 ms^−1^ [139 s ± 13.5, *t*_(59)_ = −3.492, *p* = 0.003] and linear 75 ms^−1^ profiles [158 s ± 16.1, *t*_(59)_ = 2.518, *p* = 0.038]. Time to motionless was also greater for animals undergoing the gradual 75 ms^−1^ (285 s ± 15.2) compared to the accelerated 75 ms^−1^ profile [230 s ± 12.0, *t*_(55)_ = −2.854, *p* = 0.017].

For some behavioral measures, such as urination and fore-limb movement, values were missing as not all individuals performed these behaviors (refer to Figure 2). Fore-limb movement was observed in 28 mice (38.9%) and was more likely to be observed with the faster 150 ms^−1^ rate (odds ratio = 0.18 ± 0.01, *p* = 0.002). In addition, we found that 35 mice (48.6%) urinated shortly before being deemed motionless, and C57BL/6 mice were more likely to urinate compared to Balb/c (odds ratio = 0.20 ± 0.11, *p* = 0.002). However, there was no effect of decompression shape or rate on the likelihood of urinating. For those mice that did urinate and in line with other behavioral measures, we found an effect of decompression rate where longer latencies were observed for the slower 75 ms^−1^ rate (205 s ± 16.1) compared to 150 ms^−1^ [169s ± 8.6, *t*_(21)_ = 2.321, *p* = 0.0304]. There was also a relationship between strain and profile shape, where Balb/c mice took longer to urinate (229 s ± 29.8) compared to C57BL/6 mice (136 ± 8.9) for the accelerated shape only [*t*_(21)_ = 4.009, *p* < 0.001].

### Pathological and Histological Findings

Upon recompression and removal from the chamber, death was confirmed in accordance with Schedule 1 of A(SP)A (UK) *via* a listed confirmation method (permanent cessation of the circulatory system) and a full post-mortem and macroscopic assessment were conducted (see methods and materials for full details). No concerning pathological changes were seen externally, for example we found no evidence of hemorrhage from the ears, nose, or mouth (Table 3). Internally, we found no concerning pathological changes in the joints (stifle joint) and in most organs examined including the stomach, small and large intestine where the anticipated pathological effects of pressure change were likely to be the greatest (Table 3). As expected, we found evidence of lung congestion, hemorrhage, and atelectasis both macroscopically and histologically.

**Table 3:** Summary table of findings from gross macroscopic assessment of external and internal structures and histological assessment of the lungs, middle ear and maxillary sinuses for mice undergoing decompression pooled across rate and shape given lack of statistical effects.

	**Score**					
	**0 Absent**	**1** **Minimal**	**2** **Mild**	**3** **Moderate**	**4 Marked**	**5** **Severe**
**Macroscopic assessment**
Oral cavity	48 (100)	0	0	0	0	0
External ears	48 (100)	0	0	0	0	0
Nostril/nose	48 (100)	0	0	0	0	0
Submandibular/cervical region	48 (100)	0	0	0	0	0
Skull, occipital/temporal region	48 (100)	0	0	0	0	0
Biceps femoris	48 (100)	0	0	0	0	0
Stifle (red synovial fluid)	48 (100)	0	0	0	0	0
Liver	48 (100)	0	0	0	0	0
Spleen size	38 (79.2)	10 (20.8)	0	0	0	0
Stomach	48 (100)	0	0	0	0	0
Stomach dilation	40 (83.3)	7 (14.6)	1 (2.1)	0	0	0
Small intestine	48 (100)	0	0	0	0	0
Small intestine dilation	44 (91.7)	4 (8.3)	0	0	0	0
Pancreas	48 (100)	0	0	0	0	0
Large intestine	48 (100)	0	0	0	0	0
Large intestine dilation	48 (100)	0	0	0	0	0
Kidney	48 (100)	0	0	0	0	0
Gonads	48 (100)	0	0	0	0	0
Thymus	48 (100)	0	0	0	0	0
Heart	48 (100)	0	0	0	0	0
Left lung	39 (81.3)	1 (2.1)	4 (8.3)	3 (6.25)	1 (2.1)	0
Right lung	15 (31.25)	7 (14.6)	10 (20.8)	14 (29.1)	2 (4.2)	0
**Histological assessment**
Right lung congestion	0	3 (6.25)	12 (25)	27 (56.3)	6 (12.5)	0
Right lung hemorrhage	21 (43.8)	8 (16.7)	10 (20.8)	5 (10.4)	3 (6.3)	0
Right lung atelectasis	13 (27.1)	13 (27.1)	13 (27.1)	8 (16.7)	1 (2.1)	0
Left lung congestion	0	6 (12.5)	12 (25)	27 (56.3)	3 (6.3)	0
Left lung hemorrhage	25 (52.1)	11 (22.9)	7 (14.6)	3 (6.3)	2 (4.2)	0
Left lung atelectasis	34 (70.8)	4 (8.3)	4 (8.3)	5(10.4)	1 (2.1)	0
Middle ear congestion	0	1 (2.1)	17 (35.4)	27 (56.3)	3 (6.3)	0
Middle ear hemorrhage	0	0	10 (20.8)	30 (62.5)	8 (16.7)	0
Middle ear inflammation	46 (95.8)	1 (2.1)	1 (2.1)	0	0	0
Tympanic bulla eosinophilic proteinaceous material	1 (2.1)	17 (35.4)	29 (60.4)	1 (2.1)	0	0
Maxillary sinus congestion^*^	40 (95.2)	1 (2.5)	0	0	0	0
Maxillary sinus hemorrhage^*^	41 (100)	0	0	0	0	0
Retrobulbar hemorrhage^*^	47 (100)	0	0	0	0	0

*Scores were on a 6-point scale reflecting 0 = absent; 1 = minimal; 2 = mild; 3 = moderate; 4 = marked; 5 = severe. Reported findings represent the total number of mice observed in each category and this value as a percentage of total animals analyzed presented within brackets.*

* *denotes reduced sample examined*.

**Figure 5:**
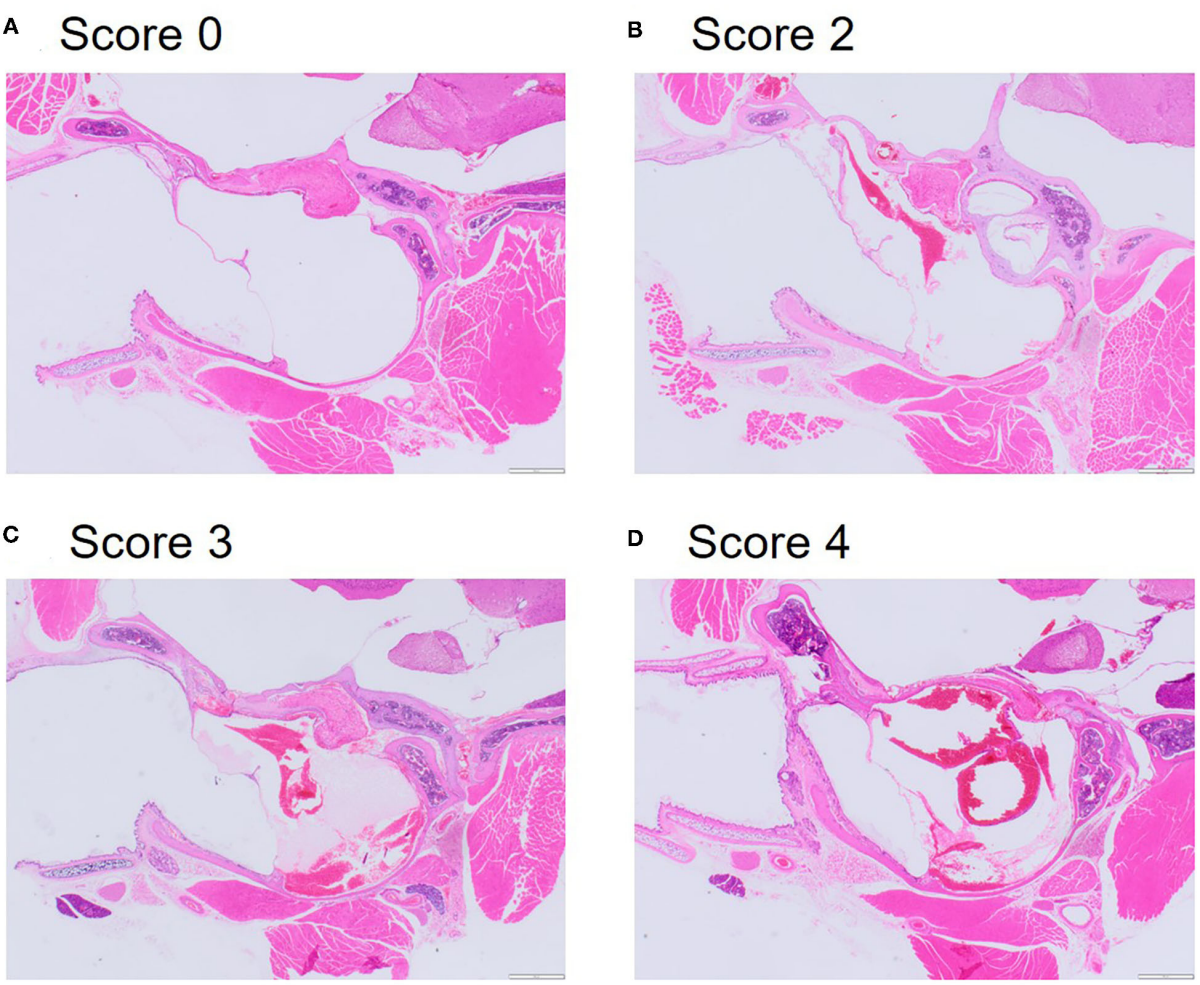
Representative microphotographs of scores for congestion and hemorrhage in the middle inner ear. **(A)** Score 0 represents absence of congestion and hemorrhage from non-decompressed control mouse. **(B)** Score 2 represents mild congestion and hemorrhage. **(C)** Score 3 represents moderate congestion and hemorrhage. **(D)** Score 4 represents marked congestion and hemorrhage. Haematoxylin and Eosin (HE) stain.

By contrast, examination of the ears resulted in cause for concern, with 56.3% of mice scoring moderate (median = 3, range: 1–4) for middle ear congestion and 62.5% of mice scoring moderate for middle ear hemorrhage (median = 3, range: 2–4). Middle ear inflammation was scored as zero except in two mice where one scored minimal (1) and another mild (2) which was noted unilaterally. Congestion and hemorrhage in the middle ear were present and consistent across all the mice (Table 3) and were most prominent in the mucoperiosteum of the ventral aspect of the tympanic bulla, through the presence of extravasated red blood cells in the cavity (Figure 5). Initially, focal discontinuity of the tympanic membrane was noted unilaterally in 8 out of 48 mice. However, additional sections confirmed 97.9% (*n* = 47) of mice had intact tympanic membranes whereby unilateral tympanic rupture was only observed in one mouse (2.1%).

We found no effect of decompression rate or shape on macroscopic or histological scores. However, we found an interaction between rate and shape when examining histological scores for middle ear congestion and hemorrhage. For congestion, within the 150 ms^−1^ rate we found a higher score for the accelerated shaped profile (mean score: 3.13 ± 0.18) compared to the gradual shaped profile (mean score: 2.25 ± 0.17, z ratio = 3.494, *p* = 0.001) but not for the linear shaped profile (mean score: 2.63 ± 0.19, z ratio = 1.93, *p* = 0.130). The accelerated 150 ms^−1^ decompression profile was again associated with higher scores for middle ear hemorrhage (mean score: 2.13 ± 0.18) compared to the linear 150 ms^−1^ profile (mean score: 1.48 ± 0.21, z ratio = 2.507, *p* = 0.033) but not the gradual shaped profile (mean score: 2.01 ± 0.19, z ratio = 1.874, *p* = 0.888).

There were no effects of strain on the majority of macroscopic or histological scores. However, we found some evidence of a strain effect in lung histology, whereby we found significantly higher mean scores for congestion and hemorrhage in Balb/c mice compared to C57BL/6 mice. This was apparent across right lung congestion (Balb/c: 3.07 ± 0.14; C57BL/6: 2.46 ± 0.15, z ratio = 3.021, *p* = 0.003), right lung hemorrhage (Balb/c: 2.84 ± 0.25; C57BL/6: 1.47 ± 0.16, z ratio = 4.614, *p* < 0.001), left lung congestion (Balb/c: 2.88 ± 0.20, C57BL/6: 2.19 ± 0.21, z ratio = 2.309, *p* = 0.021) and left lung hemorrhage (Balb/c: 2.31 ± 0.26, C57BL/6: 1.43 ± 0.17, z ratio = 2.857, *p* = 0.004).

## Discussion

This study focused on the effectiveness and reliability of gradual decompression to provide a potentially humane technique for killing laboratory mice. We are the first to conduct a robust systematic evaluation of both decompression rate and shape whilst protecting welfare through the application of general anesthesia. Our findings demonstrate that gradual decompression induces a predictable and reliable sequence of reflexive behavioral events prior to death and is highly effective in producing a non-recovery state in laboratory mice (100% kill success), with minimal pathological consequences.

In line with our expectations, we found clear effects of decompression rate on several behavioral outcomes whereby the slower rate of decompression resulted in elongated latencies to behavioral markers of death compared to the faster rate. Despite the suggestion of rodent resistance to hypoxia (37–40), death occurred in time frames that are not prohibitively time consuming. Decompression rendered mice motionless after approximately 257 ± 8.96 s (75 ms^−1^) or 214 ± 7.26 s (150 ms^−1^), whereas gradual exposure to CO_2_, which depending on the precise flow rate, is reported to occur within a range of 130–250 s (65), though we note the duration of the process is only one welfare consideration. Although the welfare consequences of decompression remain to be elucidated, this methodology has potential in terms of demand on personnel time, which remains a significant barrier to the uptake of novel methodologies and refinements (66).

Our systematic approach investigating shape and rate of the decompression profile revealed that decompression *via* an accelerated shaped profile was least susceptible to meaningful refinement *via* rate compared to gradual and linear shaped profiles. Despite reaching the final target pressure in double the time, the slower accelerated decompression profile resulted in similar latencies to key behaviors, including latency to first gasp. We also found evidence to suggest higher mean scores for middle ear congestion and hemorrhage with the faster accelerated profile compared to both linear and gradual shapes at the same rate. Consequently, our data suggest that employing slower accelerated decompression profiles would not mitigate potential negative welfare consequences such as dyspnoea and ear barotrauma in conscious mice and such profiles should be avoided.

Overall, gradual decompression and recompression were not associated with gross pathological damage in most organs examined, at least at the rates explored in this study. We only confirmed unilateral tympanic membrane rupture in one mouse (*n* = 48) undergoing decompression and subsequent recompression. However, the inconsistency of the results in the histological assessment of the additional sections suggests that the significance of the observed discontinuity of the tympanic membrane should be considered with caution. While the possibility that the observed finding represents a genuine focal rupture of the tympanic membrane remains, it cannot be completely discounted that the discontinuity could reflect an artifact related to tissue processing or microtome sectioning. Even so, legitimate concerns surrounding ear congestion and hemorrhage remain. Given that the mice were terminally anesthetized, it is difficult to infer the likely welfare impact for conscious animals. Unlike poultry, humans and other mammals including rodents have similar physiological and anatomical characteristics of the pharynx and therefore similar functioning of the eustachian tube to moderate internal ear changes in atmospheric pressure (67). Evidence from humans exposed to similar rates of decompression (simulating altitude) during military pilot training, have reported some incidences of pain and/or discomfort in the middle ear (15, 16, 68–70). In a recent study (16), comparing hypoxia symptoms and self-reported physiological effects of trapped gas between two training sessions, gastrointestinal tract discomfort was the most common physiological reaction (7.3–15.2% incidence), compared to ear blockage (2.3–13.8%), sinus blockage (4.1–4.7%) and tooth pain (0.3–0.9%). However, in addition to the relatively low incidence, it should be noted that these physiological symptoms are predominantly associated with descent from altitude, and therefore associated with recompression and not decompression (ascent) (16, 68–70). For example, Tu et al. (16) found that ear or sinus pain was a common reason to interrupt training while descending. This is significant for determining the likely welfare impact of gradual decompression for killing purposes given that decompression is occurring during the conscious phase, whilst recompression happens once the animal is rendered unconscious and in a non-recovery state. A major unavoidable limitation of this type of work is the inability to separate effects of decompression from recompression, as recompression of the chamber is always required to remove the animal. With end point pathological assessments only, it is difficult to infer the point at which hemorrhage, and congestion are occurring and full behavioral assessment for changes indicative of likely pain and/or discomfort must be studied in conscious animals. An important point to consider is that due to their unconscious state, the mice were unable to perform active behaviors in an attempt to equalize the pressure differential through motivated behaviors such as swallowing, chewing and biting. It is possible that conscious animals would be capable of greater voluntary or involuntary equalization (67, 71, 72) resulting in reduced ear barotrauma. Nevertheless, combined with reflexive behavioral results, the pathological findings support further investigation at slower overall decompression rates in conscious animals.

To comply with the principle of the 3R's, we chose to limit this initial proof of principle step to two of the most widely used mouse strains (C57BL/6 and Balb/c) and focused on male mice initially given their widespread use across biomedical research. While minor, we did observe some differences between the two strains of mice in their behavioral and pathological responses to decompression. For example, there was a greater likelihood of urination upon death in the C57BL/6 mouse strain compared to Balb/c. However, this is unlikely due to decompression but instead could be explained by differences in urinary biochemistry and output. Previous work focused on urinary biochemistry ranges across mouse strains demonstrated that C57BL/6 male mice have greater urinary output compared to male Balb/c mice, which could explain our findings (73). We also found higher mean scores for lung congestion and hemorrhage for Balb/c mice undergoing decompression compared to C57BL/6 mice. Although we cannot elucidate the mechanism behind these differences, we note that the same findings have been observed when assessing lung hemorrhage and congestion following CO_2_ killing, with more severe pulmonary hemorrhage in Balb/c mice compared to C57BL/6 mice when exposed to a pre-filled chamber and when exposed gradually (74). As such, pulmonary hemorrhage and congestion are more likely a consequence of hypoxia and/or hypercapnia, rather than a consequence of hypobaria.

Finally, the robust nature of our experimental design and highly reliable decompression chamber meant that we were able to correlate key behaviors with absolute chamber pressure. In line with our predictions, we found evidence of a cumulative hypoxic burden with profiles associated with slower inductions. We found no evidence to suggest that a particular pressure threshold was capable of eliciting a given behavioral response and instead responses relate to the preceding pressure change and the overall “hypoxic dose” over time (17). This finding is important for making inferences for subsequent work focused on determining the welfare impacts of gradual decompression using slower induction times to avoid ear barotrauma without significantly prolonging suffering and time to death.

## Conclusions

We are the first to systematically evaluate relevant responses to gradual decompression profiles that are capable of evoking a non-recovery state through hypobaric hypoxia in laboratory mice. Although valuable, the use of unconscious animals means that minimal welfare inferences can be derived from this study. However, our findings are a crucial first step, informing subsequent work in conscious mice. Subsequent work should focus on evaluating the welfare consequences of gradual decompression in conscious mice by examining responses to candidate decompression parameters, with a particular focus on identifying profiles that can prevent welfare harms associated with ear barotrauma.

## Data Availability Statement

The datasets presented in this study can be found in online repositories. The names of the repository/repositories and accession number(s) can be found below: all data will be freely available on the University of Glasgow's research online data repository: http://dx.doi.org/10.5525/gla.researchdata.1250.

## Ethics Statement

The animal study was reviewed and approved by University of Glasgow Animal Welfare and Ethical Review Body (AWERB) and project license approval was granted from the UK Home Office (TP900S002).

## Author Contributions

DM, JM, and ML devised the main conceptual ideas and acquired funding for the project. JS designed and built the decompression chamber. JC, DM, and JM designed, planned, and carried out the experiments. FM carried out post-mortem and pathological and histological assessment. JC conducted all data processing and analysis. JC drafted the manuscript with JM and DM making substantial edits in both substance and style. All authors contributed to the manuscript revision and editing.

## Funding

We would like to thank the Biotechnology and Biological Sciences Research Council (BBSRC) for funding the research project (BB/S007210/1). The Roslin Institute was funded by a BBSRC Institute Strategic Program Grant BB/P013759/1.

## Conflict of Interest

JS was employed by Livetec Systems Ltd. The remaining authors declare that the research was conducted in the absence of any commercial or financial relationships that could be construed as a potential conflict of interest.

## Publisher's Note

All claims expressed in this article are solely those of the authors and do not necessarily represent those of their affiliated organizations, or those of the publisher, the editors and the reviewers. Any product that may be evaluated in this article, or claim that may be made by its manufacturer, is not guaranteed or endorsed by the publisher.
