# Supplementary material for: Determining Candidate Hypobaric Hypoxia Profiles for Humane Killing of Laboratory Mice
Source: Front Vet Sci. 2022 Mar 23;9:834478. doi: 10.3389/fvets.2022.834478 (PMC8988232; doi:10.3389/fvets.2022.834478)
Supplement: Supplementary file 1 [file Data_Sheet_1.PDF]

Supplementary Material

1 Supplementary Figures

1.1 Decompression chamber

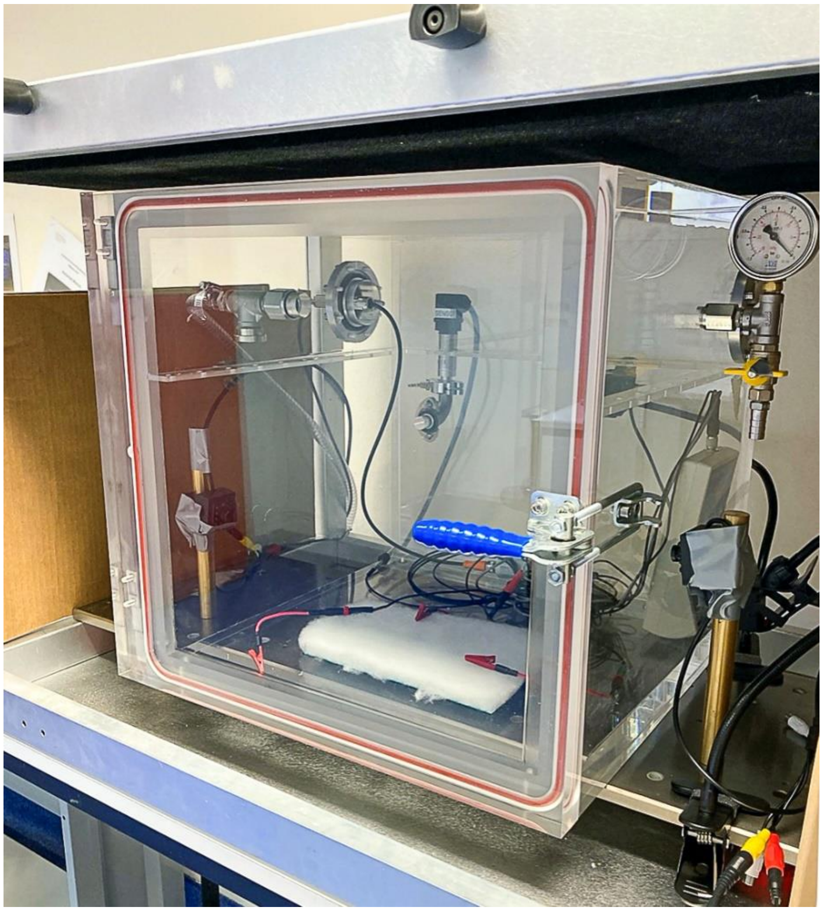

Supplementary Figure 1: Photograph of custom-designed gradual decompression chamber (Livetec Systems Ltd) and surrounding set-up for experiment, including cameras and electrocardiogram (ECG) recording equipment. Decompression is controlled by an automated programmable logic controller (PLC) system. The mouse was placed in a supine position on the white Vetbed® covered floor of the box.

1.2 Post-mortem scoring for alimentary content

Supplementary Table 1: Summary table of findings from gross macroscopic assessment of stomach fill. Scores were on a 4-point scale reflecting 0 = Empty stomach, 1= minimal amount of alimentary content, 2 = moderate amount of alimentary content, 3 = large amount of alimentary content.

Reported findings represent the total number of mice observed in each category and this value as a percentage of total animals analysed presented within brackets ().

	0<br>EMPTY	1<br>MINIMAL	2<br>MODERATE	3<br>LARGE	NOT ASSESSED
STOMACH FILL	2 (4.2%)	20 (41.7%)	23 (48%)	2 (4.2%)	4 (8.3%)

1.3 Representative microphotographs for lung histology semi-quantitative scoring

1.3.1 Congestion

1.3.1.1 Score 1 (minimal)

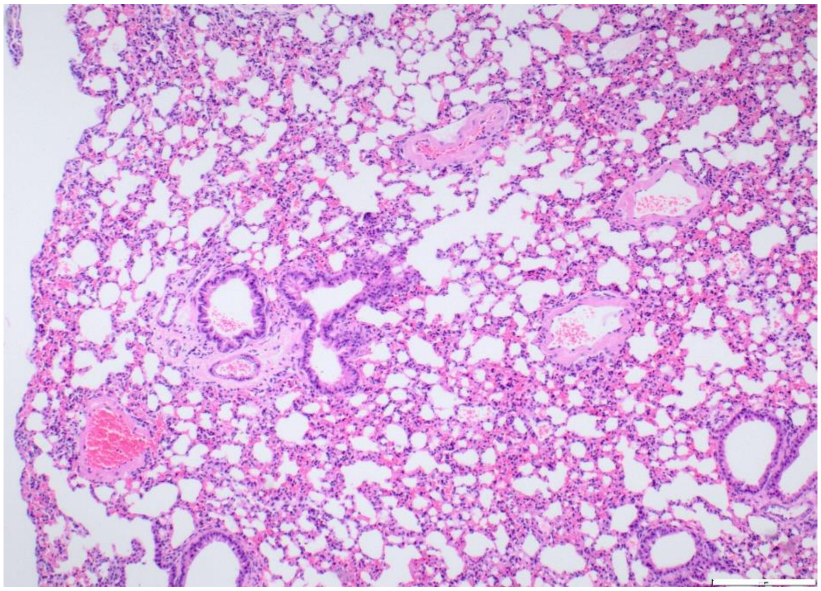

Supplementary Figure 2: Representative histologyof lung tissue following Haematoxylin and eosin staining. Example of score 1 for congestion illustrating minimal engorgement of scattered blood vessels and septal capillaries with red blood cells.

1.3.1.2 Score 2 (mild)

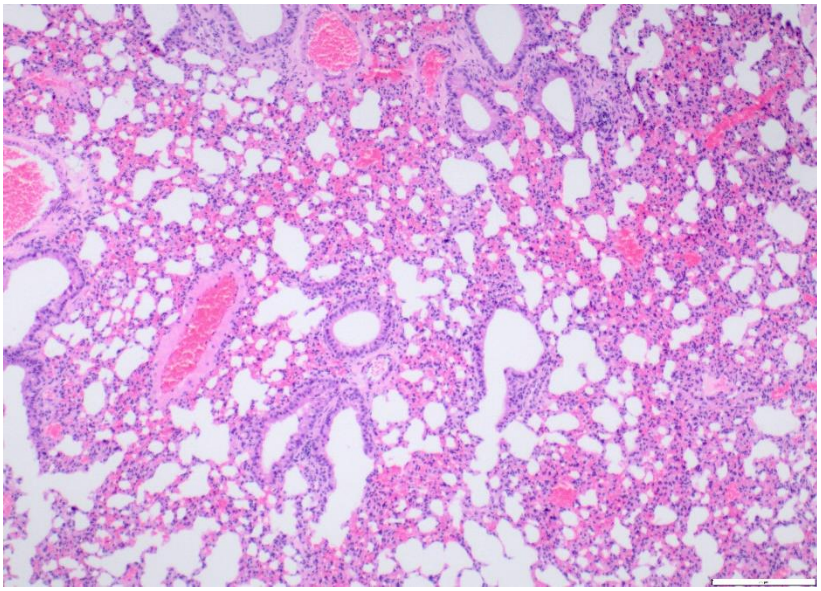

Supplementary Figure 3: Representative histology of lung tissue following Haematoxylin and eosin staining. Example of score 2 for congestion illustrating red blood cells engorging multiple vessels and septal capillaries.

1.3.1.3 Score 3 (moderate)

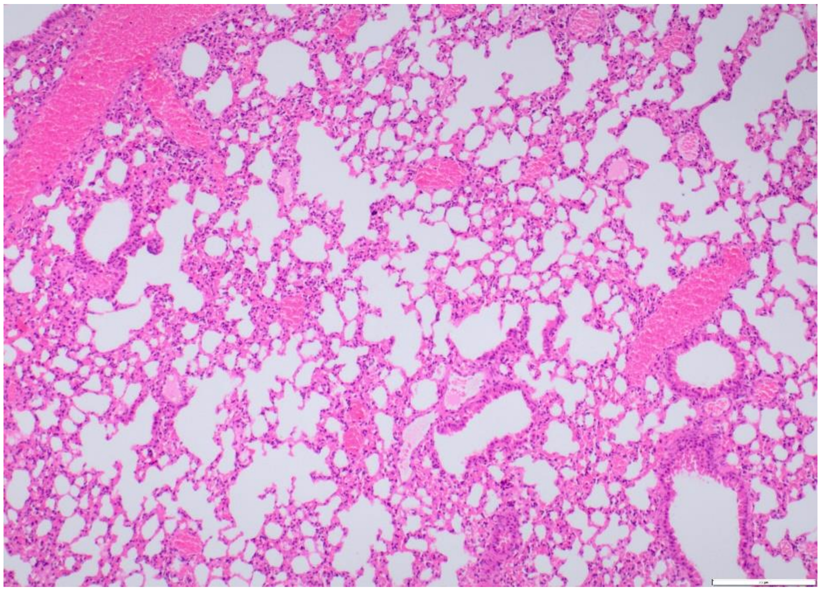

Supplementary Figure 4: Representative histology of lung tissue following Haematoxylin and eosin staining. Example of score 3 for congestion illustrating more prominent accumulation of red blood cells in the lumen of small to medium sized blood vessels and septal capillaries.

1.3.1.4 Score 4 (marked)

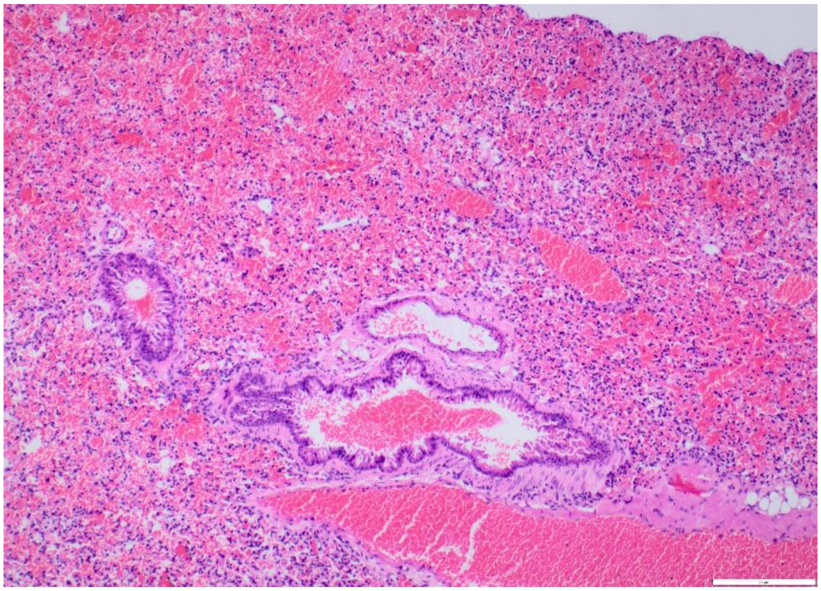

Supplementary Figure 5: Representative histology of lung tissue following Haematoxylin and eosin staining. Example of score 4 for congestion illustrating diffuse marked engorgement of blood vessels and septal capillaries with red blood cells.

1.3.2 Haemorrhage

1.3.2.1 Score 0 (absent)

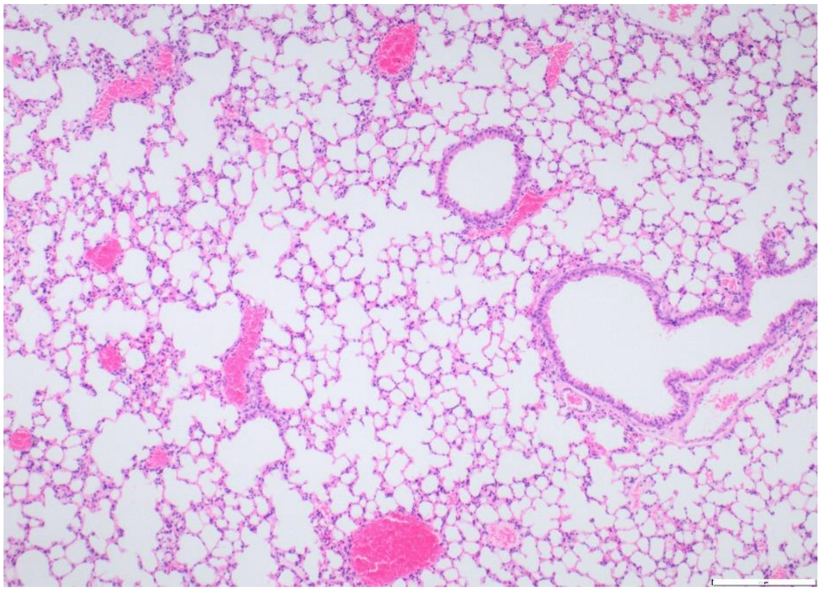

Supplementary Figure 6: Representative histology of lung tissue following Haematoxylin and eosin staining. Example of score 0 for haemorrhage illustrating no obvious accumulation of extravasated red blood cells within alveolar spaces, bronchial lumens or in the interstitium.

1.3.2.2 Score 1 (minimal)

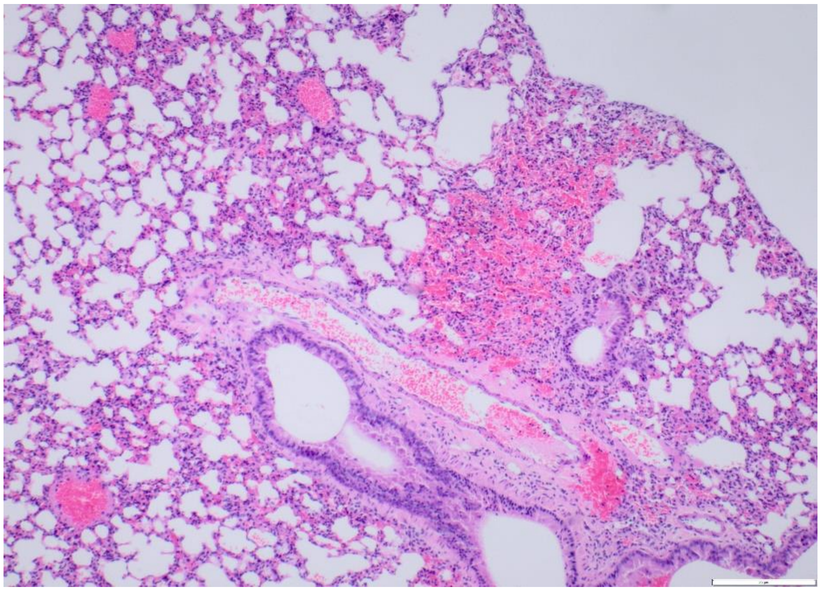

Supplementary Figure 7: Representative histology of lung tissue following Haematoxylin and eosin staining. Example of score 1 for haemorrhage illustrating minimal focal aggregates of extravasated red blood cells in alveolar spaces and with perivascular distribution.

1.3.2.3 Score 2 (mild)

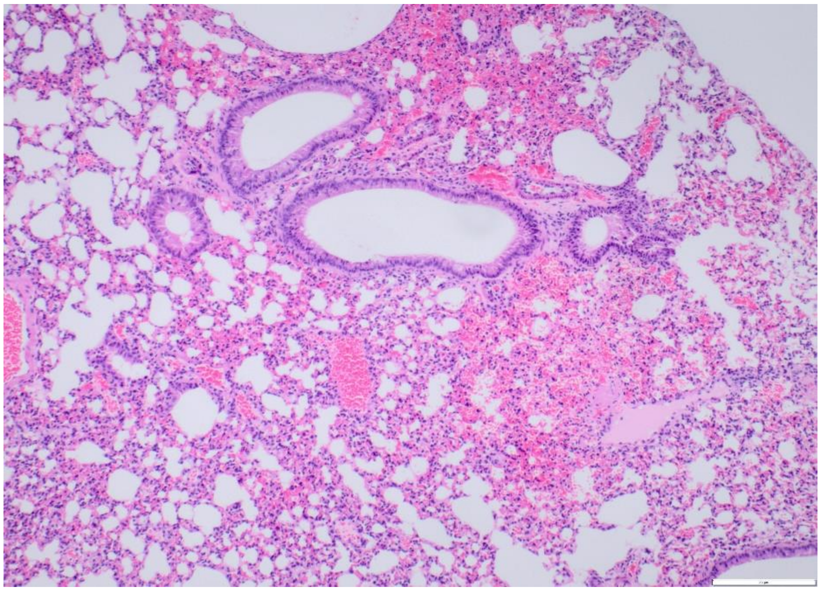

Supplementary Figure 8: Representative histology of lung tissue following Haematoxylin and eosin staining. Example of score 2 for haemorrhage illustrating multifocal small patchy areas of accumulation of extravasated red blood cells within alveolar spaces.

1.3.2.4 Score 3 (moderate)

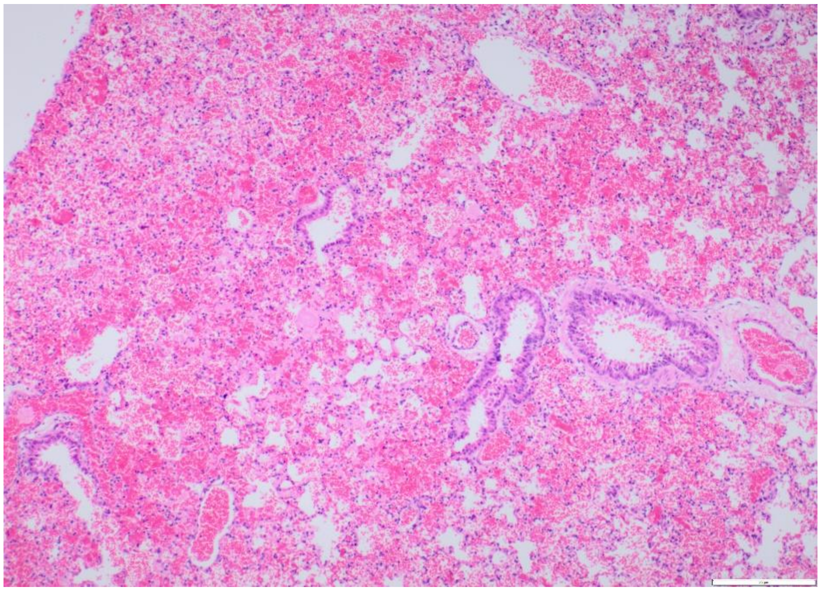

Supplementary Figure 9: Representative histology of lung tissue following Haematoxylin and eosin staining. Example of score 3 for haemorrhage illustrating local extensive moderate accumulation of extravasated red blood cells within alveolar spaces and to a lesser extent in the bronchiolar lumen.

1.3.2.5 Score 4 (marked)

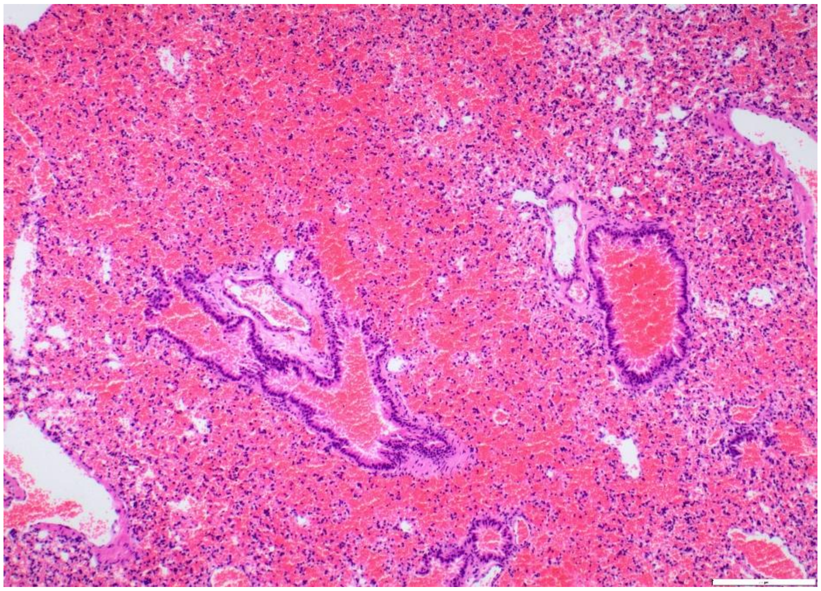

Supplementary Figure 10: Representative histology of lung tissue following Haematoxylin and eosin staining. Example of score 4 for haemorrhage illustrating marked accumulation of extravasated red blood cells within alveolar spaces and in the lumen of small bronchi and bronchioles.
